# Analysis of 37 cases of percutaneous balloon compression for primary trigeminal neuralgia: experience and outcome from a single center

**DOI:** 10.3389/fsurg.2025.1596722

**Published:** 2025-09-04

**Authors:** Hui Xu, Shilong Wang, Ganggang Wang, Jiangtao Dong, Haoxiang Xu, Wenhua Ma

**Affiliations:** ^1^ Department of Neurosurgery, The First Affiliated Hospital of Shihezi University, Shihezi, China; ^2^ Department of Interventional Surgery Center, The First Affiliated Hospital of Shihezi University, Shihezi, China

**Keywords:** primary trigeminal neuralgia, percutaneous balloon compression, puncture of foramen ovale, experience, outcome

## Abstract

**Objective:**

This study investigated the therapeutic effect and clinical experience of percutaneous balloon compression (PBC) for the treatment of primary trigeminal neuralgia (TN).

**Methods:**

This is a retrospective study. We enrolled 37 patients with symptomatic primary unilateral TN who underwent PBC and 217 patients who received microvascular decompression (MVD) at our hospital from May 2020 to May 2023. Data on demographics, surgical techniques, pain relief outcomes, and postoperative complications were collected and analyzed. The pain relief and complications of patients receiving PBC were compared to those of patients with MVD.

**Results:**

For 37 patients receiving PBC, the mean follow-up time was 12.6 months. Successful treatment was achieved in 35 cases, while 2 cases failed due to foramen ovale stenosis. Among the patients who were successfully treated, all 35 (100%) patients experienced immediate pain relief, and all developed facial numbness immediately following the procedure. At the last follow-up, 11 (31.4%) patients with facial numbness had resolved, and 24 (68.6%) patients had varying degrees of response. Masseter weakness was observed in 23 patients (65.7%), which recovered at 3 months of follow-up. No instances of intracranial hemorrhage, keratitis, diplopia, intracranial infection, or death were reported in this study. A significant association was identified between balloon compression duration, balloon shape, and pain relief outcomes, with approximately 3 min of compression and typical pear-shaped balloons achieving optimal results. Comparatively, PBC demonstrated comparable rates of complete pain relief to MVD, although MVD had lower rates of complications.

**Conclusion:**

PBC is an effective and safe minimally invasive technique for managing primary TN, demonstrating high rates of immediate pain relief. Despite being associated with transient complications, PBC allows for a rapid recovery and return to normal activities. These findings underscore the need for careful patient selection and consideration of potential postoperative complications when opting for PBC vs. MVD. Further studies should explore long-term outcomes and strategies to minimize complications associated with PBC.

## 1 Introduction

Primary trigeminal neuralgia (TN) is a prevalent cranial nerve disorder. The typical symptoms of TN include lightning-like, scalpel-like, and cautery-like pain in the facial region (1). As the disease progresses, the severity and frequency of pain attacks gradually increase. The predominant theory for the pathogenesis of primary TN is the vascular compression of the trigeminal nerve, wherein blood vessels compress the nerve roots, causing demyelination and resulting in severe pain (2, 3).

Currently, the primary treatment methods include pharmacological therapy and surgical interventions (1). Early treatment with oral carbamazepine is generally effective, but the incidence of adverse reactions, such as dizziness, nausea, and vomiting, is relatively high (3). Furthermore, with the prolonged course of the disease and increased drug dosage, the side effects become more pronounced (4). Surgical methods currently used for the clinical treatment of TN include radiofrequency thermocoagulation, microvascular decompression (MVD), and percutaneous balloon compression (PBC) (5). Radiofrequency thermocoagulation is a minimally invasive therapy that utilizes high-frequency current to create an electric field, generating heat that disrupts pain signal transmission by damaging the trigeminal ganglion, thereby achieving pain relief. Nevertheless, it carries a significant risk of recurrence post-surgery (6). MVD is the only surgical procedure targeting the underlying cause of TN. It relieves pressure from the offending blood vessels on the injured nerves. However, this technique is associated with considerable trauma and elevated surgical risk (7).

Among them, PBC is emerging as a prominent minimally invasive treatment for TN (3). The rapid and precise puncture of the foramen ovale is crucial for the successful completion of PBC surgery. During PBC, the microballoon is placed in Meckel's cave, which is then filled to create a pear-shaped structure. This configuration applies pressure to the semilunar ganglion of the trigeminal nerve, resulting in immediate relief from facial pain. The postoperative pain relief effect of PBC is substantial. In comparison to various minimally invasive or non-invasive treatment modalities, PBC is relatively straightforward, requires a short operational time, and is both minimally invasive and repeatable. However, unlike MVD, PBC is associated with a higher rate of relapse post-surgery and potential complications such as facial numbness and masseter atrophy (8). Therefore, ensuring long-term efficacy and minimizing postoperative complications remains a critical area for improvement in the surgical methodology of PBC.

This study evaluated the clinical effects and treatment experiences of 37 patients with primary TN who underwent PBC. The data on patient demographics, surgical techniques, and treatment outcomes were analyzed. Our findings may provide insights into the efficacy and safety of PBC in the treatment of primary TN.

## 2 Materials and methods

### 2.1 Study participants

The retrospective study involved 37 patients with primary TN who were admitted to the Department of Neurosurgery at the First Affiliated Hospital of Shihezi University between May 2020 and May 2023 and received PBC treatment. Inclusion criteria: 1. patients with a confirmed diagnosis of TN who consented to PBC treatment; 2. patients with significant comorbidities that precluded them from tolerating craniotomy; 3. patients who had previously taken carbamazepine but experienced insufficient pain relief; and 4. patients who experienced recurrence after MVD or other surgical interventions. Any patients meeting one or more of the above criteria were eligible for inclusion in the study. Exclusion criteria: 1. patients with cognitive impairment or mental disorders; 2. patients with secondary or bilateral TN; 3. patients with hematopoietic dysfunction or hepatic/renal impairment; 4. patients with inflammation or infection at the puncture site; 5. patients with severe arrhythmia or cardiac insufficiency; and 6. patients in poor general condition or with cachexia. To compare the surgical effects and postoperative complications between PBC and MVD, 217 patients with primary TN who were admitted to the Neurosurgery Department of the First Affiliated Hospital of Shihezi University and received MVD treatment during the same period were also included. Their clinical data, including pain relief outcome and postoperative complications such as facial numbness, weakening of masticatory muscles, intracranial infection, and keratitis, were collected. All patients underwent trigeminal nerve-specific MRI scans to exclude secondary TN caused by tumors or vascular lesions in the cerebellopontine angle and those related to multiple sclerosis. This study was approved by the Science and Technology Ethics Committee of the First Affiliated Hospital of Shihezi University (Approval no. KJX2022-084-01). Written informed consent was obtained from a legally authorized representative for anonymized patient information to be published in this article.

### 2.2 PCB procedure

All patients received oral carbamazepine before PBC. PBC was conducted under general anesthesia. In the supine position, the skin puncture site was marked on the medial side of the masseter muscle, located 2.5 cm from the lateral corner of the mouth. A trajectory was then established towards a point aligned with the medial pupillary line, situated 3 cm anterior to the external auditory canal along the zygomatic arch. Under the guidance of x-ray fluoroscopy, a 14-gauge trocar with a needle core was inserted and advanced to the outer ovoid orifice. Once the trocar contacted the foramen ovale, the core was removed, and a straight guide needle was inserted into the foramen ovale, positioned 1.5 cm distal to the inner opening. Subsequently, the 3D reconstruction of the acquired CT images of the skull base was performed to ensure that the trocar was correctly placed into the foramen ovale. The optimal view of the sella was obtained from standard lateral images of the skull, and a horizontal view was acquired to verify the accuracy of the trajectory. The guide needle was then removed, and a 4-gauge balloon catheter was inserted into the trocar, with the distal balloon positioned similarly to the guide needle. The balloon was tentatively filled with a contrast medium to observe its position and shape. If the balloon was poorly positioned or inadequately filled, the contrast agent was released immediately, the balloon was removed, and the trocar was repositioned until the desired “pear” shape was achieved. The mean duration of trigeminal nerve ganglion compression was 2 min. The contrast medium was subsequently released, followed by a repeat CT-like scan to confirm the absence of intracranial hemorrhage. Finally, the trocar was removed, and the skin puncture site was compressed for a minimum of 5 min to prevent subcutaneous hematoma.

### 2.3 Follow-up

All patients underwent routine follow-up at 1 day, 1 week, 2 weeks, 1 month, 3 months, 6 months, and 1 year after surgery. The pain relief was assessed through outpatient clinics, WeChat, telephone, etc., and graded according to the Barrow Neurological Institute (BNI) Pain Intensity Score (9). The following content was assessed, including patients' ongoing medication status, the relief of facial numbness, any occurrence of masseter muscle weakness or atrophy, corneal dryness, and the presence or remission of diplopia. The BNI pain score categorizes pain into five classes: Class I: a painless outcome (no pain and no medication); Class II: an occasional pain without the need for medication; Class III: manageable pain that can be controlled with medication; Class IV: mitigated pain but not adequately managed with medication; Class V: severe pain or no pain relief. Immediate failure was defined as postoperative TN occurring without facial anesthesia, necessitating ongoing medication for pain control. Recurrence was defined as resolution of TN for at least 3 months after surgery, followed by eventual relapse of ipsilateral TN, with or without the need for medication.

### 2.4 Data collection

The baseline clinical data (such as age, gender, etc.), intraoperative data (such as balloon volume, balloon shape, etc.), and the outcomes and complications (such as pain relief, masseter muscle weakness, facial numbness, etc.) were collected.

### 2.5 Statistical analysis

Statistical analysis was performed using SPSS Statistics 26.0. A descriptive analysis was conducted to summarize patient demographics and clinical characteristics, reporting means ± standard deviations for continuous variables and numbers (percentages) for categorical variables. Patients were stratified into two groups based on compression time: less than 3 min and 4 min or longer, or based on balloon shape: pear-shaped and non-pear shaped balloons. Kruskal–Wallis test was conducted to compare the differences in pain relief between the two groups. The surgical efficiency and complication rates between MVD and PBC for the treatment of primary TN were compared using a chi-square test. For all statistical tests, a two-tailed *P*-value of less than 0.05 was considered statistically significant.

## 3 Results

### 3.1 Demographic and baseline characteristics

The study included 37 patients diagnosed with primary TN. Their baseline information is presented in Table 1. There were 16 males and 21 females. The patients' ages ranged from 61 to 87 years, with a mean age of 68.0 ± 12.7 years. The duration of the disease varied from 0.84 to 14.0 years, with a mean duration of 3.8 ± 0.8 years. Pain was reported on the left side in 15 cases (40.5%) and on the right side in 22 cases (59.5%). The pain distribution among the branches of the trigeminal nerve was as follows: V1 branch was involved in 2 cases (5.4%), V2 branch in 11 cases (29.7%), V3 branch in 4 cases (10.9%), both V1 and V2 branches in 6 cases (16.2%), both V2 and V3 branches in 12 cases (32.4%), and all three branches (V1, V2, and V3) in 2 cases (5.4%).

**Table 1:** Baseline information of 37 patients with primary TN treated by PBC.

Variables (*N* = 37)	Patients (*N*, %)
Sex	
Male	16 (43.2%)
Female	21 (56.8%)
Age (years)	
≤65	6 (16.2%)
>65	31 (83.8%)
Lesion position	
Left side	15 (40.5%)
Right side	22 (59.5%)
Affected branches of the trigeminal nerve	
V1	2 (5.4%)
V2	11 (29.7%)
V3	4 (10.9%)
V1 + V2	6 (16.2%)
V2 + V3	12 (32.4%)
V1 + V2 + V3	2 (5.4%)
Allergy to carbamazepine	2 (5.4%)
Prior surgical treatment	6 (16.2%)
Microvascular decompression	6 (16.2%)
Radiofrequency thermo-coagulation	4 (10.9%)
Gamma knife	1 (2.7%)

TN, trigeminal neuralgia; PBC, percutaneous balloon compression.

### 3.2 The details of the PBC procedure

**Figure 1:**
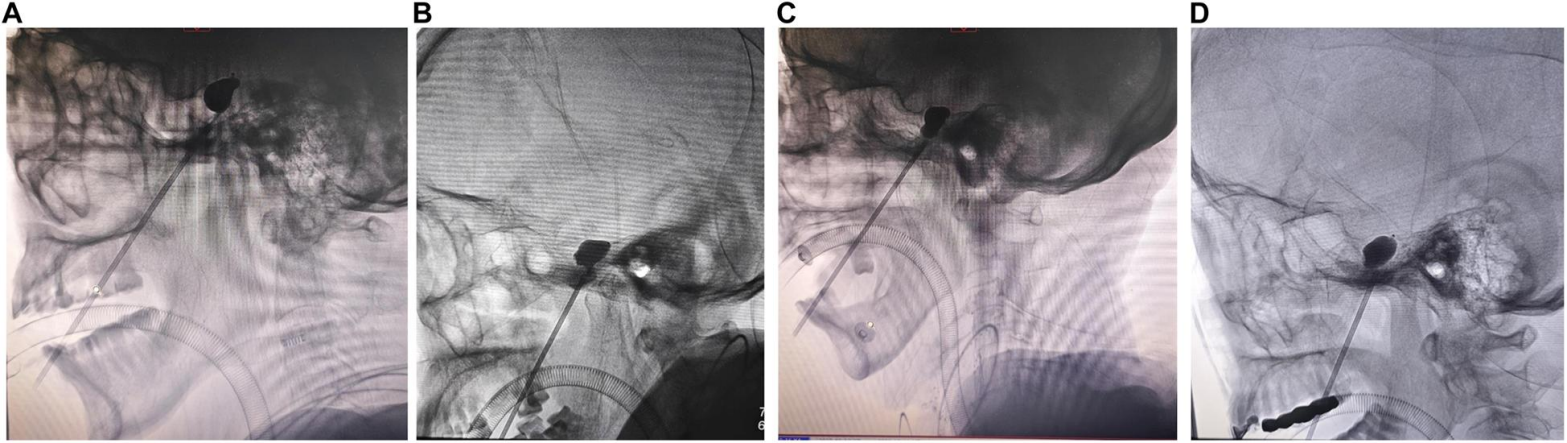
The appearance of the balloon under x-ray fluoroscopy. **(A)** Lateral skull radiographs confirm the ideal pear-shaped balloon. **(B)** Lateral skull radiographs reveal an atypical pear-shaped balloon. **(C)** Lateral skull radiographs indicate a dumbbell-shaped balloon. **(D)** Lateral skull radiographs indicate an oval-shaped balloon.

Among the 37 patients, two experienced failed punctures due to the bony ridge at the external orifice of the foramen ovale, resulting in no improvement in their condition post-surgery (5.4%) (Table 2). Among the successful cases of ovale puncture (*n* = 35), 31 patients (88.6%) exhibited a typical “pear-shaped” balloon (Figure 1A), while two patients presented with an atypical “pear-shaped” balloon (5.7%) (Figure 1B), one patient exhibited a dumbbell-shaped shape (2.8%) (Figure 1C), and one patient displayed an oval shape (2.9%) (Figure 1D) (Table 2). The balloon volume typically ranged from 0.4 to 0.7 ml (88.57%). Initially, during our implementation of balloon compression surgery, the procedure was generally performed 2–3 times, resulting in a total duration of over 3 min in 51.5% of cases, and up to 4–5 min in some patients (14.4%) (Table 2). However, as surgical techniques improved, the compression time was reduced to approximately 2 min for most patients (48.5%). In addition, among the 29 cases (82.8%) exhibiting trigeminal cardiac reflex (TCR) characterized by bradycardia (heart rate less than 60 bpm) and hypertension (blood pressure exceeding 140/90 mmHg), one case (2.8%) experienced a brief episode of cardiac arrest during the puncture of the foramen ovale. This cardiac arrest lasted less than 10 s, with the heart rate returning to normal immediately after the cessation of balloon compression. Five patients (14.4%) showed no significant changes in heart rate or blood pressure.

**Table 2:** The details of the PBC procedure in 37 patients with primary TN.

Treatment variables	Patients (*N* = 37)
Puncture	
Failed puncture	2 (5.4%)
Successful puncture	35 (94.6%)
Balloon shape	
Typical pear-shaped balloon	31 (88.6%)
Atypical pear-shaped balloon	2 (5.7%)
Dumbbell-shaped balloon	1 (2.8%)
Oval-shaped balloon	1 (2.9%)
Balloon volume (ml)	
0.3	2 (5.7%)
0.4	11 (31.4%)
0.5	13 (37.1%)
0.6	4 (11.4%)
0.7	3 (8.6%)
0.8	1 (2.8%)
0.9	1 (3%)
Compression time (min)	
2	17 (48.5%)
3	13 (37.1%)
4	3 (8.6%)
5	2 (5.8%)
Trigeminal cardiac reflex	29 (82.8%)
Transient cardiac arrest	1 (2.8%)
No changes in heart rate or blood pressure	5 (14.4%)

TN, trigeminal neuralgia; PBC, percutaneous balloon compression.

### 3.3 Treatment outcome and complications

Of the 35 patients who were successfully treated, all achieved immediate remission of TN (100%) (Table 3). We used the BNI pain intensity score to evaluate the degree of pain relief among all patients. Out of the 35 patients, 29 (82.8%) achieved complete pain relief without the use of medication (Class I) (Table 3). Three patients (8.6%) experienced occasional postoperative pain without pharmacological treatment (Class II). Postoperative pain was reported in one patient (2.8%), who required low-dose oral medication (Class III). Additionally, two patients (5.8%) experienced pain relief after surgery but required high-dose oral medication (Class IV).

**Table 3:** Outcome and complications of 35 patients with primary TN after PBC.

Outcome	Patients (*N* = 35)
Immediate relief of pain	35 (100%)
Delayed relief of pain	0 (0%)
Postoperative BNI grade	
Class I	29 (82.8%)
Class II	3 (8.6%)
Class III	1 (2.8%)
Class IV	2 (5.8%)
Class V	0 (0.0%)
Complications	
Masseter muscle weakness	23 (65.7%)
Masseter muscle atrophy	0 (0.0%)
Herpes simplex	9 (25.7%)
Diplopia	2 (5.7%)
Postoperative delirium	1 (2.2%)
Keratitis	0 (0.0%)
Intracranial infection	0 (0.0%)
Death	0 (0.0%)
Facial numbness (3 months after surgery)	
Severe	2 (5.7%)
Moderate	7 (20.0%)
Mild	15 (42.9%)
Complete resolution of facial numbness	11 (31.4%)

TN, trigeminal neuralgia; PBC, percutaneous balloon compression; BNI, Barrow Neurological Institute.

For complications, all 35 patients immediately developed mild to moderate ipsilateral numbness after surgery, which was tolerable and gradually relieved. The follow-up duration ranged from 3 to 18 months, with a mean of 12.6 months. At the final follow-up, 68.6% of patients reported persistent mild to moderate sensory decline on the affected side, though this did not interfere with their normal work or daily activities. Notably, 11 patients (31.4%) experienced complete resolution of facial numbness. Ipsilateral masseter weakness was noted in 23 patients (65.7%) (Table 3), with 19 of these cases (82.6%) recovering within 3 months post-procedure. Nine patients (25.7%) developed ipsilateral perioral herpes simplex 3 days after the operation, and recovered after one week of antiviral treatment. Two patients (5.7%) experienced diplopia, with complete symptom resolution within 3 months. Importantly, there were no instances of keratitis, intracranial infections, or fatalities reported in this study (Table 3). Pain recurred in 3 cases at the last follow-up.

Notably, we compared surgical outcomes based on balloon compression duration and shape. As shown in Table 4, among patients with compression times of less than 3 min, 30 patients (85.7%) achieved postoperative BNI scores of Class I. Conversely, among patients with compression times exceeding 4 min, only 1 patient (2.9%) achieved Class II, 2 patients (5.7%) achieved Class III, and 2 patients (5.7%) achieved Class IV scores. A statistically significant difference in pain relief was observed between the groups with compression times of less than 3 min and those exceeding 4 min (*P* < 0.05). This data suggests that the effectiveness of PBC may not be strictly linear with compression time, leading us to conclude that approximately 3 min may represent the optimal duration for balloon compression. Moreover, we observed that among patients with typical pear-shaped balloons, 29 (82.9%) had postoperative BNI scores of Class I, while 2 (5.7%) reported Class II scores. In contrast, among patients with non-pear-shaped balloons, there was 1 case (2.9%) of Class II, 1 case (2.9%) of Class III, and 2 cases (5.7%) of Class IV. Statistically, typical pear-shaped balloons achieved significantly better pain relief outcomes than non-pear-shaped balloons (*P* < 0.05), indicating that the use of a typical pear-shaped balloon during the procedure may be associated with more favorable surgical outcomes.

**Table 4:** Comparison of pain relief outcomes based on balloon shape and compression time.

Variable		Postoperative BNI grade				*P* value
		Class I	Class II	Class III	Class IV	
Balloon shape	Pear-shaped	29 (82.9%)	2 (5.7%)	0 (0%)	0 (0%)	<0.0001
	Non-pear shaped	0 (0%)	1 (2.9%)	1 (2.9%)	2 (5.7%)	
Compression time	Less than 3 min	30 (85.7%)	0 (0%)	0 (0%)	0 (0%)	<0.0001
	Greater than 4 min	0 (0%)	1 (2.9%)	2 (5.7%)	2(5.7%)	

BNI, Barrow Neurological Institute.

Kruskal–Wallis test was used for comparison.

### 3.4 One case with the adjustment of a puncture needle during surgery

**Figure 2:**
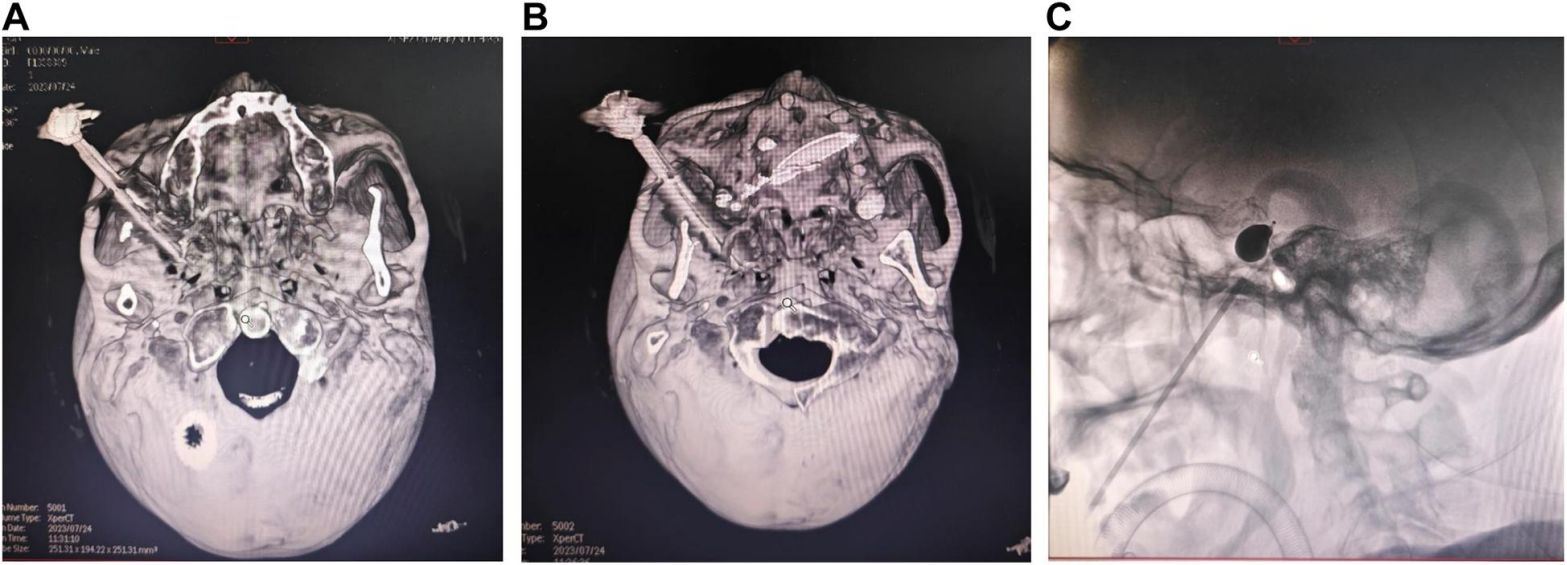
A case involving the adjustment of the puncture needle during surgery. **(A)** During the operation, the trocar failed to puncture the foramen ovale, and the 3D reconstruction of the acquired CT images of the skull base indicated that the trocar was displaced. **(B)** The trocar's position was readjusted, and a subsequent 3D reconstruction of the acquired CT images of the skull base revealed that the trocar was precisely punctured into the foramen ovale. **(C)** Following balloon inflation, the lateral cephalometric image displayed a typical pear shape.

There was one case of unsuccessful foramen ovale puncture under lateral x-ray fluoroscopy. During the operation, the trocar failed to puncture the foramen ovale. The 3D reconstruction of the acquired CT images of the skull base indicated that the trocar was displaced (Figure 2A). After readjusting the trocar's position, a subsequent 3D reconstruction of the acquired CT images revealed that the trocar was accurately punctured into the foramen ovale (Figure 2B). After balloon inflation, the lateral cephalometric image exhibited a typical pear shape (Figure 2C).

### 3.5 Comparison of the surgical efficiency and complication rates between MVD and PBC for the treatment of primary TN

Table 5 summarizes the surgical efficiency and complication rates associated with MVD and PBC for the treatment of primary TN. The surgical efficiency was assessed using the postoperative BNI grade. Among the 217 patients who underwent MVD, 180 (82.9%) achieved a postoperative BNI score of Class I, indicating complete pain relief without the need for medication. Similarly, 29 (82.9%) of the 35 patients receiving PBC reached Class I. No statistically significant differences between MVD and PBC in the rates of achieving Classes I-IV (*P* > 0.05), suggesting similar overall efficacy in pain relief.

**Table 5:** Comparison of surgical efficiency and complications between MVD and PBC.

Variable		MVD	PBC	*P*
Postoperative BNI grade	Class I	180 (82.9%)	29 (82.9%)	1.0
	Class II	18 (8.3%)	3 (8.6%)	0.76
	Class III	17 (7.8%)	1 (2.9%)	0.87
	Class IV	2 (0.9%)	2 (5.7%)	0.15
Facial numbness		5 (2.3%)	24 (68.6%)	0.003
Masseter muscle weakness		2 (0.9%)	23 (65.7%)	0.001
Intracranial infection		3 (1.4%)	0 (0%)	0.18
Keratitis		2 (0.9%)	0(0%)	0.1

MVD, microvascular decompression; PBC, percutaneous balloon compression.

Chi-square test was used for comparison.

The rates of complications, including facial numbness, masseter muscle weakness, intracranial infections, and keratitis, were further compared. Specifically, the incidence of facial numbness was significantly higher in the PBC group, with 24 out of 35 patients (68.6%) reporting this complication, compared to just 5 out of 217 patients (2.3%) in the MVD group (*P* = 0.003). Similarly, masseter muscle weakness was observed in 23 (65.7%) patients in the PBC group, while only 2 (0.9%) patients experienced this complication after MVD surgery. This difference was also statistically significant (*P* = 0.001). The incidence of intracranial infections was low in both groups, with 3 (1.4%) patients in the MVD group and none in the PBC group (*P* = 0.18). Additionally, keratitis was also infrequently reported, occurring in 2 (0.9%) MVD patients and absent in those undergoing PBC (*P* = 0.1). These findings indicate a favorable safety profile for both surgical methods.

## 4 Discussion

Initially reported by Professor Mullan in 1983, PBC is a technique that alleviates pain by mechanically compressing the sensory fibers of TN using micro balloons (10). PBC functions by employing a balloon to compress the semilunar ganglion of the trigeminal nerve, thereby blocking the nerve conduction pathway, inhibiting the release of inflammatory mediators that trigger pain, exerting analgesic effects, and attenuating the inflammatory response (11). In comparison to various minimally invasive or non-invasive treatment options, PBC presents a relatively simple surgical procedure, requires a short operative duration, results in minimal surgical wounds, and offers high surgical repeatability (12). Furthermore, the postoperative complications associated with PBC have not significantly impacted patients' daily lives. Consequently, PBC has gained increasing popularity among clinicians in recent years (13). In our study, 35 patients experienced immediate relief from postoperative pain symptoms, representing 94.4% of the cohort. Among the 32 patients evaluated for pain relief, 29 achieved a BNI level of 2 or below, while three patients experienced poor outcomes, accounting for 5.6%.

The Meckel's cave, where the semilunar ganglion of the trigeminal nerve is located, is pear-shaped. Therefore, in most literature reports (2, 14), during PBC surgery, the presence of a typical pear-shaped lateral x-ray image post-balloon inflation indicates the correct placement of the balloon within Meckel's cave, thereby suggesting that the patient is likely to experience favorable therapeutic outcomes (15). However, there are subtle anatomical differences among the individuals. In this study, among the 35 patients who were successfully punctured, balloon morphology was typical pear shape in 31 cases, atypical pear shape in 2 cases, and dumbbell shape in 1 case, all achieving relatively positive therapeutic results post-surgery. We categorized patients into two groups based on balloon shape: typical pear-shaped and non-pear-shaped balloons, to assess their impact on pain relief outcomes. Our findings indicate that patients with typical pear-shaped balloons during surgery were more likely to achieve postoperative BNI scores of Class I. This suggests that a typical pear-shaped balloon is associated with favorable surgical outcomes, consistent with previous findings (16). Additionally, to ensure the accuracy of foramen ovale puncture, we conducted both lateral x-ray fluoroscopy and 3D reconstruction of the acquired CT images of the skull base. Foramen ovale puncture was first performed under lateral x-ray fluoroscopy, and then the accuracy of the puncture was verified in a 3D reconstruction of the acquired CT images of the skull base. In one case, repeated foramen ovale puncture under lateral x-ray fluoroscopy was unsuccessful, with the deviation of the puncture point confirmed by the 3D reconstructed image. The puncture was subsequently adjusted based on this deviation, resulting in a successful outcome after the second adjustment.

In this study, we found that there were 30 patients with a balloon compression time of 2–3 min, accounting for 85.6%, 3 patients with 4 min, and 2 patients with more than 5 min. A careful review of patient data indicated that the 5 patients who experienced compression times exceeding 3 min were all from the early stage of this procedure. This may have occurred due to insufficient expertise during the initial implementation of the operation and a lack of confidence during the balloon compression, which led to the attempt to achieve a better therapeutic effect by extending the duration of compression. However, with advancements in operational techniques and the accumulation of experience, subsequent procedures have successfully maintained balloon compression times within the targeted duration of 3 min. To compare the effect of compression time on pain relief outcome, we stratified patients into two groups: less than 3 min and 4 min or longer. The results revealed that for patients with compression time less than 3 min, the majority of them had postoperative BNI scores at class I, indicating that 3 min may be an appropriate duration for balloon compression. Based on our follow-up observations, we identified 32 cases with effective therapeutic outcomes in this study, accounting for 91.4%. Notably, 85.6% of patients who experienced balloon compression for less than 3 min also reported favorable results, suggesting that prolonged compression is not a critical determinant for achieving these outcomes. On the contrary, all 7 patients still had more than moderate facial numbness symptoms in the follow-up three months after surgery. This indicates that extended balloon compression results in more pronounced complications of postoperative facial numbness, with no improvement in surgical effectiveness. It has been reported that the duration of balloon compression is positively correlated with the facial numbness of patients after PBC (17). Consistently, our study found that all 35 patients experienced facial numbness after the procedure. At the three-month follow-up, 2 patients (5.7%) continued to have severe numbness, 7 patients (20%) reported manageable numbness symptoms, 15 patients (42.8%) experienced mild numbness, and 11 patients (31.4%) were free from numbness. Nonetheless, we observed that some degree of facial numbness often serves as an indicator of the significant effectiveness of PBC surgery, and symptoms tend to diminish or resolve over subsequent follow-ups, which is consistent with other studies (18, 19).

TCR is a characteristic reaction during PBC surgery. It is essentially a complex brainstem reflex and can be induced by any stimulation involving the sensory branch of the trigeminal nerve (20). The most common manifestations of TCR are bradycardia and elevated blood pressure. Therefore, TCR can induce catastrophic consequences for patients with pre-existing cardiovascular conditions. In our study, we found that among the 35 patients who were successfully treated, 30 patients had TCR, accounting for 85.7%, and one patient had cardiac arrest, which was quickly relieved by the release of balloon pressure, leading to the restoration of normal heart rhythm within approximately 10 s Notably, some patients' blood pressure increased to over 180 mmHg, and the postoperative blood pressure gradually recovered about 10 min after the decompression. Based on our observations regarding TCR, we recommend that, before balloon compression, communication with the anesthesiologist is essential to maintain a heart rate above 60 beats per min and blood pressure below 120/80 mmHg.

Additionally, we compared the surgical efficacy and complication rates between MVD and PBC for treating primary TN. Notably, both surgical strategies demonstrated meaningful effectiveness in achieving pain relief, with comparable rates of complete pain relief as indicated by postoperative BNI Class I scores. This suggests that surgical intervention, whether through MVD or PBC, provides substantial benefits for patients suffering from this debilitating condition. However, significant differences in complication rates highlight critical considerations when choosing between these two techniques. The higher incidence of facial numbness and masseter muscle weakness associated with PBC indicates that while it is an effective treatment, it comes with unique risks that may concern patients and healthcare providers alike. The findings align with existing literature (21) that has documented these complications as common outcomes of PBC, enhancing the notion that careful patient selection and preoperative counseling are essential. On the contrary, the MVD approach presented a lower risk of these specific complications, making it a favorable option for patients who prioritize minimizing postoperative side effects. Moreover, the low incidence of intracranial infection and keratitis across both groups further confirmed the overall safety of these surgical options, instilling confidence in their application. Given these observations, clinicians are encouraged to weigh the risks and benefits of each procedure, considering patient preferences and specific clinical scenarios. Patients who are concerned about facial numbness or masseter muscle weakness may prefer MVD, while those prioritizing a potentially less invasive approach may still consider PBC despite its associated risks.

This study has several limitations, including the retrospective sample migration, single-center design, small sample size, and short follow-up period. To address these limitations, future research should expand the sample selection and implement long-term follow-up observations. Additionally, future studies should adopt more rigorous study designs to yield more objective and accurate research results.

In summary, we thoroughly discussed the key aspects of the PBC procedure, focusing on the relationships among balloon compression duration, postoperative efficacy, and complications, as well as specific techniques for ovale puncture. Our experience with PBS for primary TN demonstrates that it is a highly effective surgical intervention with a notable rate of immediate pain relief and a favorable safety profile. Although patients experienced common side effects such as facial numbness and masseter weakness, these complications were transient for most, allowing patients to resume normal activities relatively quickly. The findings also highlighted the significance of balloon compression duration of approximately 3 min and typical pear-shaped balloons in ensuring optimal therapeutic outcomes. Notably, PBC demonstrated comparable rates of complete pain relief to MVD, but higher complication rates than MVD, particularly regarding facial numbness and masseter weakness, necessitating careful patient selection and thorough preoperative counseling. We believe that the experience of this study can provide some guidance for the application of future PBC surgeries in the treatment of patients with primary TN, particularly those who are not suitable candidates for more invasive surgical options.

## Data Availability

The raw data supporting the conclusions of this article will be made available by the authors, without undue reservation.
